# Functional interface micromechanics of 11 en-bloc retrieved cemented femoral hip replacements

**DOI:** 10.3109/17453674.2010.480938

**Published:** 2010-05-21

**Authors:** Kenneth A Mann, Mark A Miller, Nico Verdonschot, Timothy H Izant, Amos Race

**Affiliations:** ^1^Department of Orthopaedic Surgery, SUNY Upstate Medical University, Syracuse, NYUSA; ^2^Radboud University Nijmegen Medical Centre, Nijmegenthe Netherlands; ^3^Syracuse Orthopaedic Specialists, Crouse Hospital, Syracuse, NYUSA

## Abstract

**Background and purpose:**

Despite the longstanding use of micromotion as a measure of implant stability, direct measurement of the micromechanics of implant/bone interfaces from en bloc human retrievals has not been performed. The purpose of this study was to determine the stem-cement and cement-bone micromechanics of functionally loaded, en-bloc retrieved, cemented femoral hip components.

**Methods:**

11 fresh frozen proximal femurs with cemented implants were retrieved at autopsy. Specimens were sectioned transversely into 10-mm slabs and fixed to a loading device where functional torsional loads were applied to the stem. A digital image correlation technique was used to document micromotions at stem-cement and cement-bone interfaces during loading.

**Results:**

There was a wide range of responses with stem-cement micromotions ranging from 0.0006 mm to 0.83 mm (mean 0.17 mm, SD 0.29) and cement-bone micromotions ranging from 0.0022 mm to 0.73 mm (mean 0.092 mm, SD 0.22). There was a strong (linear-log) inverse correlation between apposition fraction and micromotion at the stem-cement interface (r^2^ = 0.71, p < 0.001). There was a strong inverse log-log correlation between apposition fraction at the cement-bone interface and micromotion (r^2^ = 0.85, p < 0.001). Components that were radiographically well-fixed had a relatively narrow range of micromotions at the stem-cement (0.0006–0.057 mm) and cement-bone (0.0022–0.029 mm) interfaces.

**Interpretatation:**

Minimizing gaps at the stem-cement interface and encouraging bony apposition at the cement-bone interface would be clinically desirable. The cement-bone interface does not act as a bonded interface in actual use, even in radiographically well-fixed components. Rather, the interface is quite compliant, with sliding and opening motions between the cement and bone surfaces.

## Introduction

There has been long-standing interest in the scientific evaluation of retrievals from both failed and well-functioning total joint replacements to improve our understanding of fixation and loosening mechanisms. In terms of component fixation, histological and morphometric analysis of bony ingrowth between implant and bone for press-fit implants and apposition between cement and bone for cemented implants have been investigated (Charnley 1979, Draenert 1981, Jasty et al. 1991, Maloney et al. 2002). To elucidate the role of mechanics in biological response, the influence of motion at the implant interface on the resulting biological structure that forms around the implant has been studied, usually using an animal model with carefully controlled implant motions (Pilliar et al. 1986, Soballe et al. 1992, Jasty et al. 1997, Greenfield and Bechtold 2008). One challenge with all of these studies is that it is difficult to infer the actual micromechanics at the bone-biomaterial interfaces from functional loading of the implants because direct measurement at the material interface is not possible.

A recent study in our laboratory investigated the micromechanics of small samples of the femoral cement-bone interface taken from post-mortem retrievals (Miller et al. 2010). In that study, we found that the cement-bone interface was very compliant (low stiffness) when loaded in tension and compression and also had low strength when compared to laboratory-prepared cement-bone specimens. One limitation of that experiment was that a small section of interface was isolated for testing purposes; this could have disrupted individual trabeculae and pedicles of cement, which in turn could affect cement-bone connectivity and load transfer across the interface. A further limitation was that mixed-mode loading conditions with shear, tension, and compression were not considered; these may be important modes of load transfer in vivo.

In the present study, we torsionally loaded whole transverse sections of en-bloc retrieved cemented femoral hip replacements and combined this with an imaging approach that allowed the quantification of the micromotion along the stem-cement and cement-bone interface. In this way, the displacement response of the construct to torsional loading was quantified globally, at all observable locations along the interfaces. Furthermore, the morphology of the interfaces and cement mantle was quantified and related to their micromechanics. Using this approach, we addressed 3 research questions: (1) how much micromotion is present at the stem-cement and cement-bone interfaces for en-bloc retrieved cemented total hip replacements?; (2) can the magnitude of interface motion be related to interface morphology at both interfaces?; (3) what is the difference in micromotion response between radiographically well-fixed and loose components?

## Methods

### Specimen preparation

Femora, implanted with cemented hip components, were retrieved from 11 donors at autopsy (Table 1) through the anatomical donor programs at SUNY Upstate Medical University and the University of Alabama at Birmingham. Donations to the anatomical donor programs were generally made between 1 and 2 days of death, and frozen at –20ºC prior to tissue harvest. Age, sex, cause of death, implant manufacturer, and years in service were documented. Antero-posterior and medial-lateral radiographs were obtained following removal of soft tissue and the qualitative radiographic status (definitely loose, possibly loose, not loose) was assessed by the contributing orthopedic surgeon (TI). The proximal femora were potted at the mid-diaphysis along the long axis of the femur and a transverse section of the cemented stem construct with a thickness of 10 mm was created just below the lesser trochanter for each donor bone using a water-irrigated saw. A high-resolution reflected white light image (5.7 μm/pixel) was captured of each transverse section to document the morphology of the section. The surface roughness (Ra) of the stem was determined post-experiment using a surface profilometer. Use of vacuum mixing was assessed by observation of mid-mantle porosity on the sectioned surfaces. Specimens lacking mid-mantle porosity were most likely vacuum-mixed.

**Table 1.** Donor information for the eleven cemented implants

A	B	C	D	E	F	G	H	I
A ^**a**^	76	F	2	1.29	Y	N	Breast cancer	Modular calcar replacement (BP) – Zimmer
B ^**a**^	76	F	5	3.19	Y	N	Breast cancer	Precision long stem (BP) – Howmedica
C	87	F	0.9	2.94	N	N	Cardiac arrest	Perfecta (BP) – Wright Medical Technology
D	88	M	0.2	5.73	Y	N	Cardiac arrest	Perfecta PDA calcar replacement (HE) – Wright Medical Technology
E	80	F	20+	0.99	N	N	Cardiac arrest	Müller Curved (TH) – JRI Ltd.
F	77	F	NA	0.87	N	P	Adeno-carcinoma	Cemented F Series (BP) – Implex
G	93	F	NA	6.85	Y	N	Renal insufficiency	Omnifit w/proximal rough surface (BP) – Osteonics
H	92	F	6	0.73	Y	N ^**b**^	Renal insufficiency	Endurance (TH) – Depuy Orthopaedics
I ^**a**^	85	F	8	0.75	Y	P	Bacterial endocarditis	Versys cemented (TH) – Zimmer
J ^**a**^	85	F	8	2.5	Y	Y	Bacterial endocarditis	Versys cemented (TH) – Zimmer
K	67	F	14	1.3	Y	N	Alzheimer’s disease	Harris precoat (TH) – Zimmer

^**a**^ Two pairs of donor bones (A-B and I-J) were from bilateral hip replacements.

^**b**^ Donor bone H had an extensive cement mantle fracture, most likely from a fall, resulting in a mid-shaft fracture sustained 5.5 years after the total joint replacement procedure.

A Donor

B Age

C Sex

D Years in service NA: Not avaiable

E Stem roughness (Ra, μm)

F Vacuum-mixed

G Radiographically loose N: not loose, P: possibly loose Y: definitely loose

H Cause of death

I Implant type – Manufacturer TH: Total hip replacement HE: Hemiarthroplasty BP: Bipolar hemiarthroplasty

### Mechanical testing

**Figure 1.**
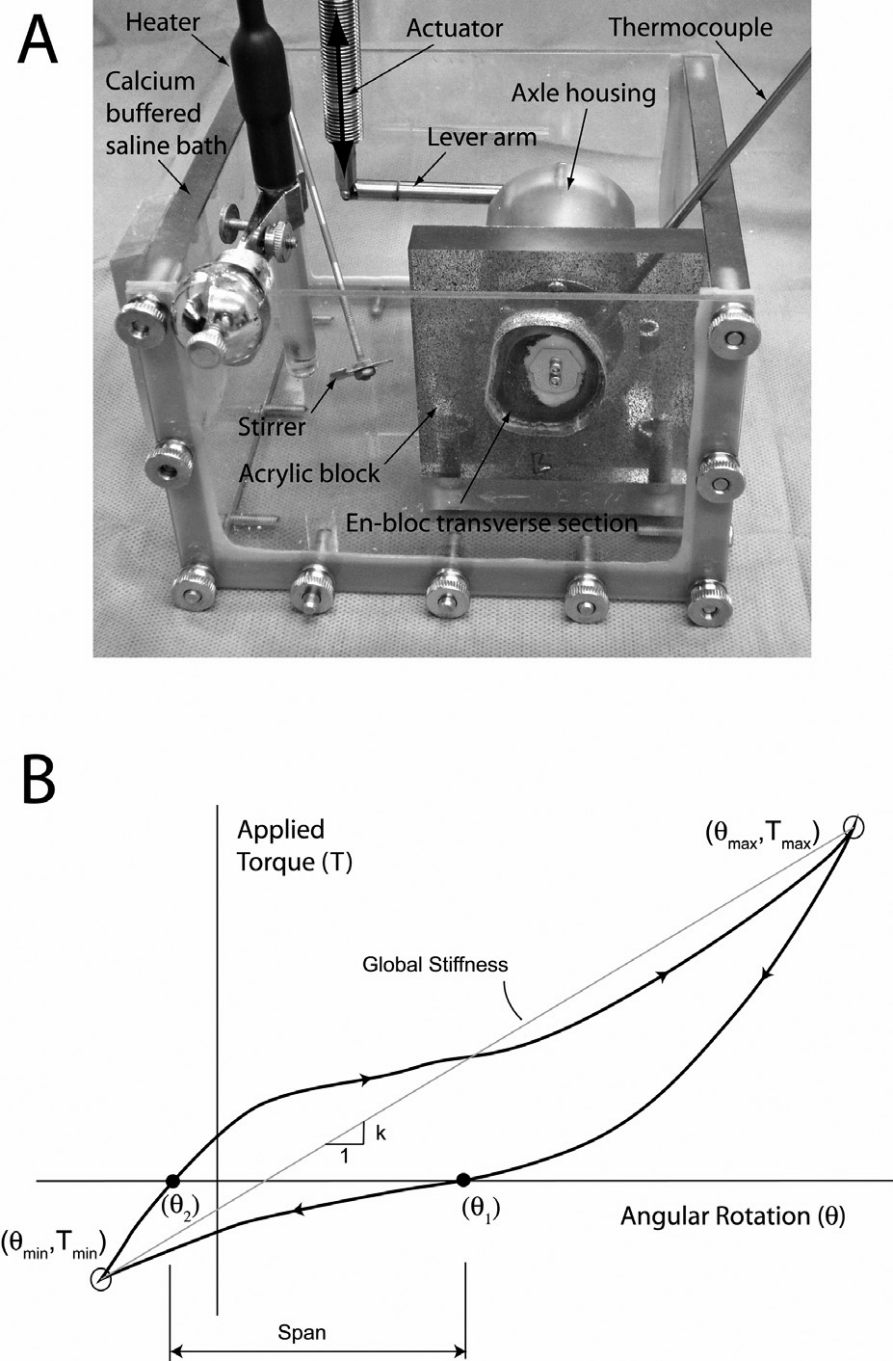
The apparatus for applying torque to the en-bloc transverse section of the cemented femoral hip components (panel A) consisted of a pure torque applied to the stem via an axle that was secured to the stem with two small screws. Torque was applied to the axle via a lever arm driven by the test frame actuator. The bone was glued to a polycarbonate plate, which was fixed to the base of the test frame. The global response was recorded (panel B) as applied torque vs. angular rotation with global stiffness and span as the primary outcome measures. Tmax represents peak retroversion and Tmin represents peak anteversion applied to the section.

The transverse sections were fixed to a custom-machined polycarbonate block using an acrylic adhesive on the periosteal bone surface (Figure 1A). A pure torsion loading device consisting of a torque rod in an axle housing with ceramic bearings was attached to the stem. The stem was mechanically loaded via a 72-mm lever arm attached to a mechanical test frame actuator (Instron 1122; Instron, Norwood, MA). To facilitate optical displacement measurements, the specimens were speckled with black enamel aerosol paint, creating optical texture on the surface of the en-bloc transverse section. Testing was conducted in a clear polycarbonate container filled with calcium-buffered saline maintained at 37ºC.

We chose a torsional loading regime for this project because out-of-plane torques during gait and stair ascent are known to generate high hoop stresses in the femoral component of total joint replacements (Bergmann et al. 1995). Torque was applied to the en-bloc sections in displacement control (0.13 deg/sec) to limits of 0.73 Nm in retroversion and 0.22 Nm in anteversion. 3 preconditioning cycles were applied and images of the surface (see below) were captured during the last loading cycle. The global response of the en-bloc specimen was defined by excursion of the loading arm (converted to rotation angle) and applied force (converted to torque). The global torsional stiffness (Nm/deg) was calculated from the slope of the torque-rotational angle response (Figure 1B). The span of the torque-rotation response, defined as the difference in rotation angles at zero torque, was used as an indicator of overall laxity of the specimen section.

### Optical displacement measurement of interface micromotion

**Figure 2.**
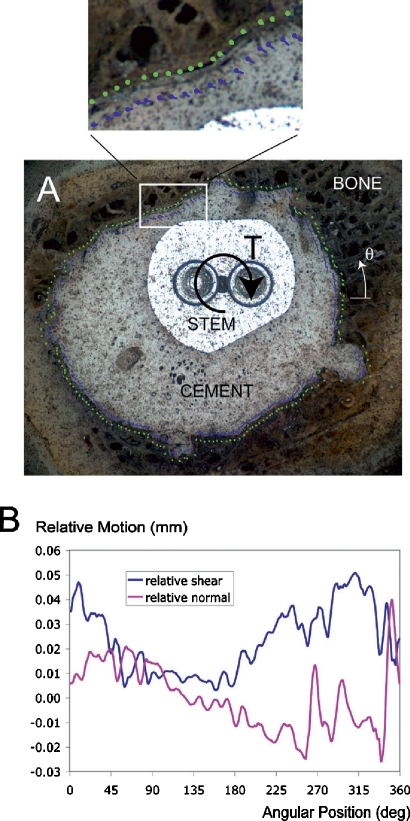
A digital image correlation technique was used to quantify displacements adjacent to the interfaces (panel A). This example from donor bone C shows motion of the cement (blue vector lines) and bone (green vector lines) along the cement-bone interface. These were further decomposed into relative normal motion and shear motion at the interface as a function of angular position (panel B). Angular position (Θ) increases in a counter-clockwise direction with 0 degrees starting from a horizontal line, as shown in panel A.

We used a digital image correlation (DIC) technique to quantify the micromotion along the stem-cement and cement-bone interfaces (Figure 2). A CCD camera with telecentric lens (0.021–0.025 mm/pixel resolution) was used to document motion of the stem, cement, and bone during mechanical loading at a rate of 4 Hz. Software code was written to determine the relative motion at 180 sampling locations (2-degree angular position increments) along the stem-cement and cement-bone interfaces. The sampling locations were placed at a distance of 0.25 mm from the interface to prevent errors in the DIC sampling at the material discontinuities. The RMS error of the DIC system was 0.0011–0.0013 mm as determined in preliminary tests with controlled input displacements. Because micromotion was calculated as differences in displacement between two DIC locations, potential errors could double (0.0022–0.0026 mm).

The interface micromotions were calculated at the extrema of loading (as shown in Figure 1) and the total excursion of interface micromotion between maximum retroversion and maximum anteversion is reported here. Both normal interface and shear components of interface micromotion were calculated, and the total interface micromotion was calculated as the vector sum of the normal and shear components. Cumulative frequency distributions of interface micromotions were generated for each specimen (based on the 180 sampling points) and the median micromotion was used as the primary outcome measure of interface motion.

### Quantification of interface gap thickness

**Figure 3.**
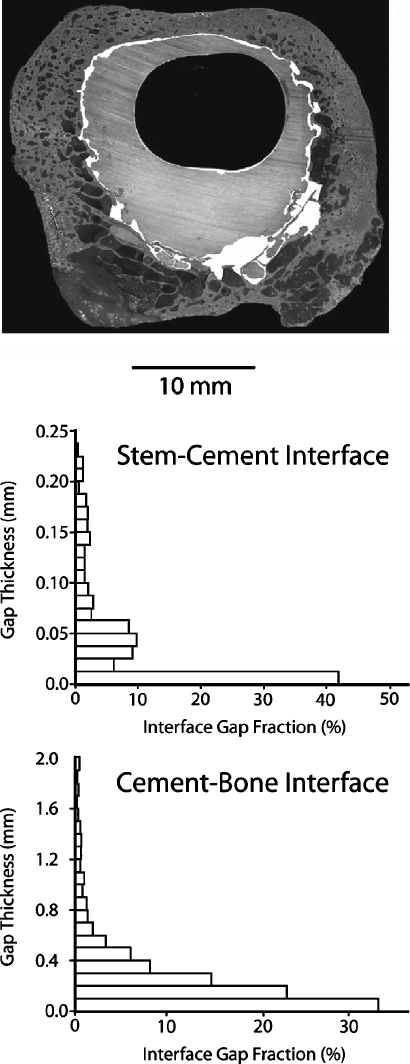
Gaps at the stem-cement and cement-bone interface gaps are indicated in white for donor bone B. Image processing was used to determine the frequency distribution of gap thickness for the interfaces.

The distribution of gap thickness was calculated for the stem-cement and cement-bone interfaces using a multi-step image processing approach (Figure 3). A contour line was drawn at each interface to delineate the material boundaries (stem from cement and cement from bone) and gaps at the interface were identified and filled manually for each specimen image. A distance map filter in Image Pro (Media Cybernetics, Bethesda, MD) was used to generate a list of gap thicknesses. The fraction of apposition, defined here as the cumulative frequency of gaps less than 11 microns thick (2 pixels in width), was used as a measure of apposition at the interfaces for each specimen. Median gap thickness was calculated as an additional measure of the population of interface gaps.

### Statistics

The within-specimen normal motion to shear motion relationship of the stem-cement and cement-bone interfaces was evaluated using linear regression. Linear regression analysis was also used to determine if there was a within-specimen relationship between motion at the stem-cement interface and cement-bone interfaces. A paired t-test was used to determine if there was more motion at the stem-cement interface or the cement-bone interface. Regression analysis was also used to determine whether there was a relationship between interface apposition and micromotion at the stem-cement and cement-bone interfaces.

## Results

**Figure 4.**
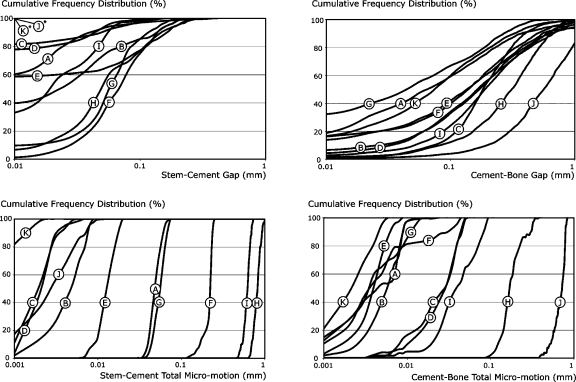
Cumulative frequency distributions are shown for gap measurements (top) and interface micromotion (bottom) for the stem-cement and cement-bone interfaces. The letters correspond to the donor bones as listed in Table 1. Note that donor bones K and J had stem-cement gaps of less than 0.01 mm for 100% of the interface.

There was a wide range of responses to mechanical loading of the 10-mm-thick en bloc transverse specimens (Table 2) with torsional stiffness (70–3,721 Nm/deg) and span (0.003–9.54 deg) varying by several orders of magnitude. Micromotion at the stem-cement and cement-bone interfaces also varied widely (see Table 2 and Figure 4). For the 11 samples, there was no within-specimen correlation between median micromotion at the stem-cement interface and the cement-bone interface (r^2^ = 0.04). Cumulative frequency distributions of interface micromotion (Figure 4) illustrated that the differences between specimens were greater than the variations in micromotion within a particular specimen. Total median micromotion at the interfaces spanned 3 orders of magnitude depending on the test specimens for both stem-cement (0.0006–0.832 mm) and cement-bone (0.0022–0.727 mm) interfaces.

**Table 2.** General mechanical response of the stem-cement (S-C) and cement-bone (C-B) interfaces to torsional loading and morphology measures for the eleven test specimens. Total micromotion is the vector sum of the shear and normal components of interface motion. The data presented in the table for micromotion and gap thickness for the eleven en-bloc specimens are the median response for each test parameter

	Mean (n = 11)	Standard deviation	Range
*Mechanical Response Parameters*			
Global torsional stiffness (Nm/deg)	1,369	1,160	70–3,721
Torsional span (deg)	2.06	3.19	0.003–9.54
Median S-C shear micromotion (mm)	0.162	0.285	0.0003–0.813
Median S-C normal micromotion (mm)	0.021	0.044	0.0003–0.129
Median S-C total micromotion (mm)	0.165	0.290	0.0006–0.832
Median C-B shear micromotion (mm)	0.087	0.207	0.0019–0.694
Median C-B normal micromotion (mm)	0.021	0.044	0.0007–0.148
Median C-B total micromotion (mm)	0.092	0.216	0.0022–0.727
*Morphology Parameters*			
Minimum mantle thickness (mm)	0.55	0.58	0–1.7
S-C gap thickness < 11 μm fraction (%)	52.0	36.3	1.4–100
C-B gap thickness < 11 μm fraction (%)	10.4	10.2	0.4–32.5
Median S-C gap thickness (mm)	0.032	0.027	0.0–0.08
Median C-B gap thickness (mm)	0.187	0.146	0.045–0.57

When comparing normal micromotion at the interface to shear micromotion, there was a strong linear correlation for the stem-cement interface (normal-to-shear ratio: 0.15; r^2^ = 0.89, p < 0.001) and the cement-bone interface (normal to shear ratio: 0.23; r^2^ = 0.99, p < 0.001). These results show that with torsional- (shear-) type loading applied to these specimens, motion at the interfaces was mixed-mode (a combination of normal and shear motion) with less but proportional normal micromotion when compared to shear micromotion.

Gaps at the stem-cement interface were thinner (p = 0.004, paired t-test) than those at the cement-bone interface (Table 2). The distribution of gaps at the stem-cement interface was also of a narrower range than for the cement-bone interface (Figure 4). 2 of the donor specimens (K and J) had no measurable gaps at the stem-cement interface (i.e. 100% apposition). In contrast, the cement-bone interface with the greatest apposition fraction had only 33% contact between cement and bone.

**Figure 5.**
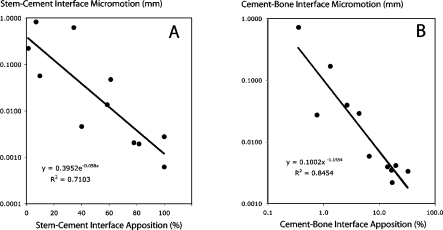
An exponential relationship was found between apposition fraction of the stem-cement interface (cumulative frequency of gaps less than 11 μm (0.011 mm) in thickness) and stem-cement interface total micromotion (panel A). There was a Power-law relationship between apposition fraction of the cement-bone interface (cumulative frequency of gaps less than 11 μm (0.011 mm) in thickness) and cement-bone interface total micromotion (panel B). The total micromotion data presented here represent the median of the vector sum of normal and shear components of relative motion.

Specimens with a high stem-cement apposition fraction (cumulative frequency of gaps less than 11 μm) had low amounts of micromotion at the stem-cement interface (Figure 5A). The relationship between apposition fraction and micromotion was non-linear and fitted an exponential form (r^2^ = 0.71, p < 0.001) for the stem-cement interface. A similar inverse relationship between high cement-bone apposition fraction (cumulative frequency of gaps less than 11 μm) and low micromotion was also determined for the cement-bone interface (Figure 5B). In this case, we found a power-law relationship (r^2^ = 0.85, p < 0.001) between apposition fraction and micromotion at the cement-bone interface.

**Figure 6.**
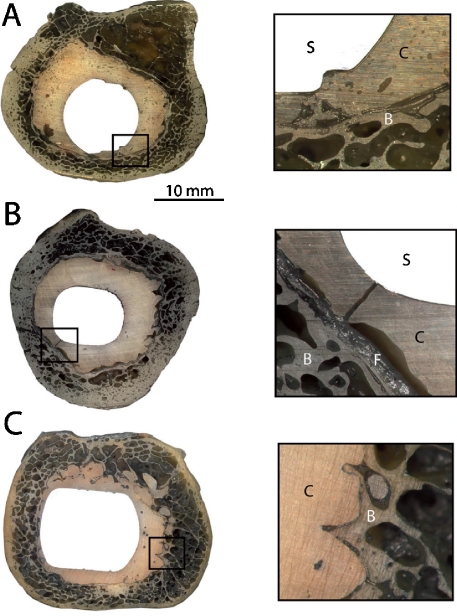
Transverse sections of 3 en-bloc retrievals illustrating a possibly loose component (panel A, donor bone F), a cement fracture associated with excessive motion and fibrous tissue formation (panel B, donor bone H), and a radiographically well-fixed component (panel C, donor bone K). In the inset images, the stem (S), cement (C), bone (B), and fibrous tissue (F) are indicated.

There were 7 well-fixed components based on radiographic criteria (Table 3): 2 that were possibly loose, and one that was definitely loose. One additional specimen (donor bone H) appeared to be radiographically well-fixed but had a large longitudinal fracture of the cement mantle, resulting in excessive stem-cement motion (Figure 6). This cement fracture was most likely due to a right mid-shaft periprosthetic fracture of the femur sustained from a fall, 5.5 years after the total hip replacement procedure was performed. The composite total micromotion (stem-cement plus cement-bone) was much larger for the loose, possibly loose, and fracture specimens than for the well-fixed components. The well-fixed components had stem-cement motions ranging from 0.0006 to 0.057 mm and cement-bone motions ranging from 0.0022 to 0.029 mm.

**Table 3.** Micromotion behavior of the stem-cement and cement-bone interfaces stratified by radiographic status

Radiographic status ^**a**^	Median stem-cement total micromotion (mm) ^**b**^	Median cement-bone total micro-motion (mm) ^**c**^	Median composite (S-C + C-B) total micromotion (mm) ^**c**^
Loose (n = 1)	0.0028	0.73	0.73
Possibly loose (n = 2)	0.43 (0.29) [0.23–0.63]	0.022 (0.026) [0.0035–0.040]	0.45 (0.31) [0.23–0.67]
Fracture (n = 1)	0.83	0.17	1.00
Well-fixed (n = 7)	0.018 (0.024) [0.0006–0.057]	0.011 (0.011) [0.0022–0.029]	0.029 (0.021) [0.0028–0.061]

^**a**^ Number of specimens in each category is indicated (n)

^**b**^ Mean and standard deviation (in parentheses) are indicated along with range (in square brackets).

^**c**^ Composite micromotion is the sum of stem-cement and cement-bone components.

## Discussion

**Figure 7.**
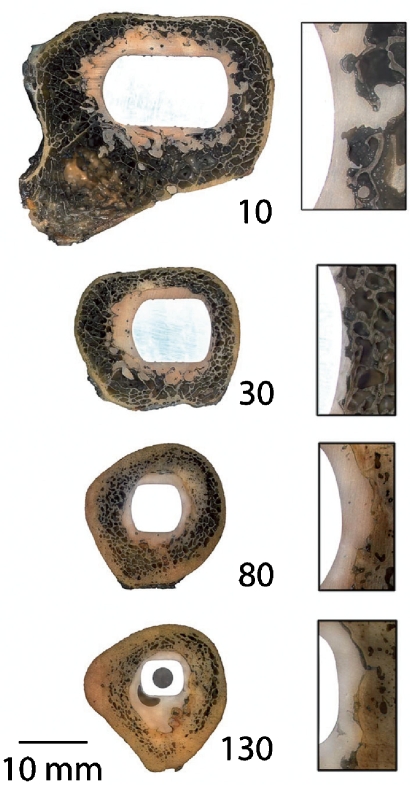
Transverse sections from the radiographically well-fixed (donor bone K) component at 10, 30, 80, and 130 mm below the stem collar. Section “10” is at the level of the lesser trochanter and section “130” is at the distal tip of the stem. Inset images illustrate the stem, cement, and bone at the lateral side of the construct. Section “30” was evaluated in this study.

In answer to the first research question, our observations showed that functional loading of en-bloc retrieved cemented femoral hips resulted in a wide range of measurable micromotions at the stem-cement and cement-bone interfaces. For the stem-cement interface, there were several cases where stem-cement motion was at or below the limits of detection of micromotion, suggesting that some specimens exhibited bonding at that interface. For the cement-bone interface, there were detectable micromotions along the entire cement-bone interface for all of the test specimens. This finding suggests that, following implantation, the cement is not bonded to the bone but rather behaves as a compliant interface with motion between the cement and bone components. Even the most well-fixed implant (donor bone K, Figure 7) had measurable, but small, micromotions along all of the cement-bone interface. Of interest was that this donor bone had a Harris Precoat stem design with 14 years of service. The precoat was intact and considerable force was required to remove the stem from the cement mantle to measure surface roughness. While there have been reports of early loosening of Precoat designs (Dowd et al. M1998), this particular specimen was well-fixed.

The second research question concerning the relationship between micromotion and interface morphology was affirmed in that gap distributions at the stem-cement and cement-bone interface were strongly related to micromotion at the interface. Correlations between migration of components from fatigue loading and interface morphology have also been reported for laboratory-prepared constructs (Race et al. 2006, Mann et al. 2007). However, it is likely that the laboratory-prepared specimens do not correctly mimic the clinical situation, particularly with regard to the cement-bone interface (Miller et al. 2010). The third research question addresses the question of how much motion is present at the stem-cement and cement-bone interfaces for radiographically well-fixed components as compared to loose components. Statistical analysis was not performed here because of the relatively small sample size of the loose component group and the wide range of responses for the loose group. As a whole, it appears that well-fixed components can have measurable micromotions at the stem-cement interface, which is consistent with the notion that the stem-cement interface often behaves as a frictional non-bonded interface (Verdonschot and Huiskes 1997). The cement-bone interface from radiographically well-fixed en-bloc retrievals function with micromotions in the 2–30 μm range and that these micromotions are evident over the majority of the interface. Interestingly, 2 of the possibly loose specimens had large stem-cement micro-motions along with small cement-bone micromotions, suggesting that either the cement-bone interface is not always loose (even with radiographic evidence) or that the interface was more loose at other regions of the construct.

### Limitations

There are several limitations to this study. The loading regime used only included pure torsion. Loading in other planes, in particular axial loading, could change load transfer due to wedging that might occur due to the taper of the stem in the cement and the cement in the bone. The process of cutting with water irrigation could have disrupted poorly organized connective tissue between the cement and bone. Well-organized fibrous tissue was evident in some of the test specimens, but with the preparation techniques used, it is not possible to know whether less organized tissue was displaced during processing. While a reasonable number of donor bones (11) were available for our study, it is clear that the wide variability in responses we found cannot provide a complete understanding of the distribution of mechanical response or morphology from the total joint population.

Unfortunately, medical histories were not available for most of these donor bones. In addition, details of the surgical techniques used to perform the implantations were not available. For example, it is not known whether all were primary hip replacements or if femoral canals were broached or reamed. There is evidence that vacuum mixing was used in 8 of 11 donor bones and that a distal plug was used in at least 10 of the cases. One en-bloc retrieval was sectioned at the distal tip of the stem, so that it is not known if a distal plug was used in that case. The radiographs used to detect radiolucencies did not include soft tissue, and were also obtained as contact radiographs; this probably resulted in an improved ability to visualize interface gaps than would be possible with clinical radiographs.

We evaluated only one section of the construct for each of the en-bloc retrievals. Sections above or below the level tested could have a different mechanical response to loading. To test for this, we performed torsional loading experiments on sections from 3 of the donor bones proximal to the sections presented in our study. The more proximal sections were more torsionally stiff (donor bone B: 3,094 vs. 2,302 Nmm/deg; bone D: 2,374 vs. 1,979 Nmm/deg; donor bone I: 221 vs. 111 Nmm/deg), but the difference between donor bones was greater than within the donor bones. Additional testing would be useful to map micromotions along en-bloc specimens from the stem collar to the distal tip of the stem.

### Relevance of the torsional loading model

Torsional loading due to posteriorly directed forces at the femoral head has been shown to contribute to high cement mantle stresses using finite element models (Stolk et al. 2002). In addition, stem retroversion has been shown to be the primary mode of implant migration along with axial subsidence in clinical radiographic stereometric analysis (RSA) studies (Nivbrant et al. 1999). Laboratory-based studies of stair-climbing loading using cemented femoral hip components have resulted in similar findings; stem retroversion and axial subsidence are the two primary modes of motion for the stem relative to the bone (Race et al. 2005). Based on these findings, axial torque is considered to be an important load component between the stem and bone in total joint replacement.

In our study, the center of rotation of torsional load was applied at centroid of the stem. However, it is possible that transverse displacements combined with rotation could effectively move the center of rotation from the centroidal axis of the stem. To provide an estimate of how much the center of rotation might move in a fully-cemented construct, we analyzed data from 6 degree-of-freedom measurements made during stair-climbing loads applied to laboratory-prepared cemented femoral components (Race et al. 2005). These motion measurements were made between the stem and the bone using a high-resolution linear variable differential transducer (LVDT) system at the mid-stem level. After 300,000 cycles of stair-climbing loading, the center or rotation moved, on average 0.73 mm laterally and 0.07 mm posteriorly, using cyclic motion data. These findings suggest that the center of rotation could remain close to the stem centroid. But, there may also be instances—due to differences in stem shape, gap distributions at the stem-cement and cement-bone interfaces, and cement mantle distributions—that could alter the center of rotation in the retrieval specimens used in our study. Changes in the actual center of rotation would affect the local micromotions at the stem-cement and cement-bone interfaces.

The magnitude of torsional loading we applied to the transverse sections was 0.74 Nm (acting over a 10-mm section). In a full construct, torsional loads of 10–17 Nm for slow gait (3 km/h) and 25–35 Nm for stair climbing have been measured using an instrumented hip system (Bergmann et al. 1995). If loaded uniformly along the length of the stem with nominal length of 150 mm, an equivalent sectional load would be in the range of 0.7–1.1 Nm for gait and 1.6–2.3 Nm for stair climbing. Of course, loading is not uniform along the length of the stem, but the experimental sections were taken from the level of the lesser trochanter and substantial load transfer between implant and bone is likely at that level (Mann et al. 1997). Thus, the micromotions quantified here are in the range of nominal gait loading but do not represent the maxima that would be experienced in vivo. It should be noted that because of the non-linear torque-rotation behavior of these interfaces (see Figure 1B), larger loading magnitudes would not result in proportional increases in micromotion in general.

### Relation to previously published work

The relationship between mechanical loading, micromotion, local morphology, and the biological response at the implant-bone interface has been studied using a number of animal models. The implants most used were fabricated from metal or preformed PMMA. The upper limit of micromotion to allow for osseointegration in these model systems has been reported to be in the 20–120 μm range, but this depends greatly on the characteristics of the local environment (Pilliar et al. 1986, Jasty et al. 1997, Greenfield and Bechtold 2008). Using a computational mechano-biological approach, Andreykiv et al. (2008) simulated the time course of tissue differential at the bone-implant interface while varying the implant surface roughness, gap thickness, and interface micromotion. They predicted a higher rate of bone ingrowth into rougher interfaces, cases with micromotion of less than 75 μm, and thicker interfaces. The finding that interfaces with greater gap thickness (100 μm compared to 50 μm) were more favorable in terms of bony ingrowth for the same amount of initial micromotion is most likely due to the lower physical stimuli for the larger gap condition in terms of fluid flow and shear distortion. This resulted in osteoblast differentiation rather than fibroblast differentiation in this model. While it is difficult to translate the findings of these studies to the work performed here because the amount of micromotion was not controlled, it is interesting to note that most of the micromotions at the interface for our retrieval specimens was less than 100 μm, with about half having cement-bone micromotions of less than 10 μm.

### Clinical relevance

The strong relationship between micromotion and interface morphology for both stem-cement and cement-bone interfaces suggests that steps to minimize interface gaps at the stem-cement interface and enhanced bony apposition at the cement-bone interface would be desirable for long-term viability of these joint replacements. Given the extensive data showing long-term success of cemented femoral stems (Malchau et al. 2005), it is intriguing to find evidence that they may all exhibit debonding through opening and sliding motion at the cement-bone interface—i.e. that they may all be, if only slightly, loose. It is likely that the compliance of the cement-bone interface is the result of remodeling of the bone. Further experimental and finite element modeling work will be needed to understand how changes to the cement-bone and stem-cement interface as documented here would be manifested in terms of our understanding of load transfer. It is also possible that implant designs that compress the cement-bone gap with loading, such as tapered stem designs, may reduce micromotion at the cement-bone interface.

3 of the 11 constructs we evaluated had hand-mixed cement, as evidenced by extensive mid-mantle porosity. Because of the small sample size, it was not possible to statistically assess the effect of vacuum mixing on the morphology or micromotion measurements. The 3 hand-mixed cases had gap distributions and micromotion distributions that fell well within the range of responses found for vacuum-mixed cases (see donor bone C, E, and F in Figure 4). This suggests that factors other than mid-mantle porosity may be affecting the in vivo response of the constructs. This is consistent with the view that mid-mantle porosity reduction may not have a major effect on the clinical performance of cemented femoral hip components (Ling and Lee 1998, Malchau et al. 2000, Janssen et al. 2005, Hernigou et al. 2009).

Advances in surgical cementing technique resulting in a cement mantle that is complete with a centralized stem and interlock between the cement and bone would appear to represent best practices for long-term success of cemented femoral components. In total joint registries, second- and third-generation cementing techniques have been shown to dramatically improve outcomes (Herberts and Malchau 2000). To support these clinical studies, post-mortem retrieval analysis has been used to more fully document the surgical cementing technique and function of the joint replacements following in vivo service. Recently, Bishop et al. (2009) analyzed the mantle morphology of 214 cemented hip replacements and they found debonding of the stems in 82% of the cases and thin cement mantles in 74% of the cases. These findings suggest that the goal of achieving an optimal mantle may not be realized in many cases. Ideally, retrieval analysis would be combined with details of the surgical procedure including canal preparation technique (Ioannidis et al. 2005), magnitude of cement pressurization, and cement mixing technique (Hernigou et al. 2009), along with serial X-ray series during clinical follow-up. The high-resolution imaging techniques developed for this study, coupled with careful quantification of both interface morphology and micromechanics, could be useful tools for improvement of our understanding of how cemented implants function.
